# Complete mitochondrial genome sequence of *Myrmecophaga tridactyla* from Brazilian Savanna

**DOI:** 10.1080/23802359.2018.1481785

**Published:** 2018-06-12

**Authors:** Ana Luiza Lemos Queiroz, Anderson Oliveira do Carmo, Yan Kalapothakis, Ana Paula Vimieiro Martins, Evanguedes Kalapothakis

**Affiliations:** Laboratory of Biotechnology and Molecular Markers, Biological Sciences Institute, Federal University of Minas Gerais, Belo Horizonte, MG, Brazil

**Keywords:** Complete mtDNA, next-generation sequencing, giant anteater

## Abstract

Myrmecophaga tridactyla, popularly known as giant anteater, is a member of Xenarthra magnorder which is under the threat of extinction. Herein, we describe the complete mitochondrial genome of M. tridactyla. The circular DNA molecule is 16,546 bp long, contains 13 protein-coding genes, two rRNA genes, 22 tRNA genes, and a non-coding Control Region of 1110 bp. All protein-coding genes are on the heavy strand, except for Nd6. Ten of the 13 PCGs contained an ATG start codon.

*Myrmecophaga tridactyla* (Linnaeus, 1758), commonly known as giant anteater, is a member of Xenarthra magnorder. It is currently found in a wide variety of habitats in South and Central America, and is currently listed as “Vulnerable” in the IUCN Red List of Threatened Species. According to Miranda et al. (2014), factors that led to its decline include dietary specificity, low reproductive rates, large body size, habitat loss, road kills, and illegal hunting. During 2013–2014, *M. tridactyla* was one of the species at the top of the list of illegally slaughtered animal carcasses reported by the environmental military police (Chagas et al. 2015).

This study utilized a female *M. tridactyla* individual rescued by the environmental military police in Ituiutaba, state of Minas Gerais, Brazil (18° 55′24.55′′ S/49° 25′18.61′′ W). Blood samples were provided by the Brazilian Environmental Agency (IBAMA) with the following licensing from the competent authorities: SISBIO: 56471-1, IEF: 024/2016, and CEUA-UFMG 37/2017. Genomic DNA was extracted following the phenol-chloroform protocol of Sambrook and Russell (2006) and a DNA sample was stored at Mammals DNA and tissue Bank LBEM at the Federal University of Minas Gerais (deposit code: M002146). A genomic library was constructed and sequenced using a paired-end 300-bp strategy on a MiSeq system (Illumina, San Diego, CA). *De novo* assembly was performed using Mira 4.0 (San Francisco, CA) (Chevreux et al. 1999). The complete mitochondrial DNA of *M. tridactyla* (GenBank accession number MH142215) was found to be a unique, circular, 16,546 bp long with maximum coverage of 373 reads. The mtDNA displayed a GC content of 39.2%.

The mitogenome was annotated with MITOS (Bernt et al. 2013) and verified with ExPASy (Gasteiger et al. 2003). Annotation revealed 13 protein-coding genes (PCGs), two rRNA genes, 22 tRNA genes, and a noncoding Control Region (D-loop).

Genes in *M. tridactyla* were arranged similarly to those in a typical vertebrate mitogenome. All gene sequences were also analyzed manually. Protein-coding genes commonly had ATG as start codon (10 PCGs), followed by ATA. Eight of the 13 PCGs contained a TAA stop codon.

Eight of the 22 tRNAs and one PCG (Nd6) were encoded on the light (L) strand. The remaining genes were encoded on the heavy (H) strand. We found 12 overlapping regions.

A *M. tridactyla* mitogenome of an individual from French Guiana already exists on GenBank (accession number: NC_028572.1), which is 46 bp shorter than the mtDNA sequenced in our laboratory. This difference is mainly in the D-loop. Differences in the nucleotide composition were evaluated using the CGView Comparison Tool (University of Alberta, Canada) (Grant et al. 2012) and BlastN (National Library of Medicine, Maryland, USA) (Altschul et al. 1990) that revealed >99% of identity between the two sequences.

Phylogenetic analyses were conducted using MEGA version 6.06 (MEGA Inc., Englewood, NJ) (Tamura et al. 2013). Phylogeny reconstruction was done using the maximum likelihood method with the Nearest-Neighbor-Interchange heuristic method. Due to its hypervariability, the D-loop region was excluded from phylogenetic analysis (Gonder et al. 2007). The result is consistent with other molecular phylogenies (Delsuc et al. 2002) (Figure 1).

**Figure 1.**
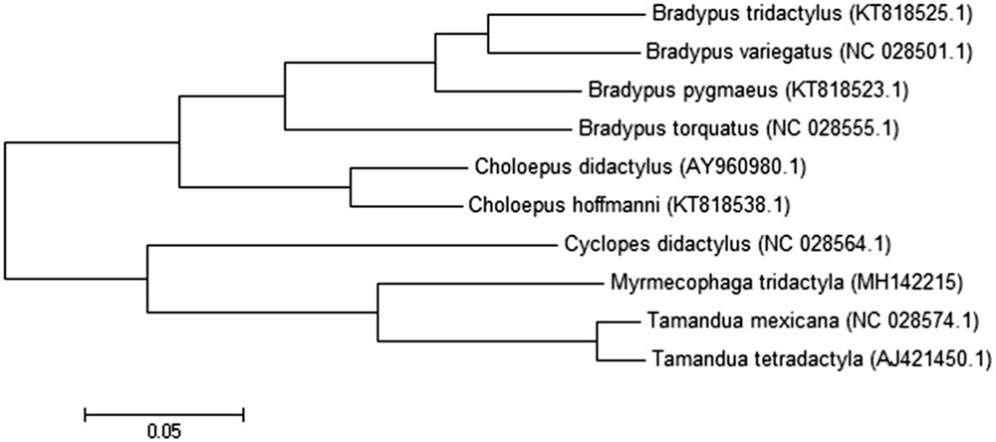
Molecular phylogeny of *Myrmecophaga tridactyla* mitochondrial genome, based on maximum likelihood (ML). Phylogenetic analyses were conducted with *Cyclopes didactylus* (GenBank accession number: NC_028564.1), *Tamandua mexicana* (NC_028574.1), *Tamandua tetradactyla* (AJ421450.1), *Choloepus didactylus* (AY960980.1), *Choloepus hoffmanni* (KT818538.1), *Bradypus pygmaeus* (KT818523.1), *Bradypus tridactylus* (KT818525.1), *Bradypus torquatus* (NC_028555.1), and *Bradypus variegatus* (NC_028501.1).
